# Characterizing the features and course of psychiatric symptoms in children and adolescents with autoimmune encephalitis

**DOI:** 10.1007/s00406-021-01293-5

**Published:** 2021-07-17

**Authors:** R Rosello, B Girela-Serrano, S Gómez, B Baig, M Lim, S Taylor

**Affiliations:** 1 Division of Psychiatry, Imperial College, London, UK; 2 Division of Psychiatry, King’s College, London, UK; 3 South London and Maudsley NHS Trust, London, UK; 4 Children’s Neurosciences Centre, Evelina London Children’s Hospital, London, UK; 5 Department Women and Children’s Health, School of Life Course Sciences (SoLCS), King’s College London, London, UK

**Keywords:** Encephalitis, Anti-N-methyl-D-aspartate receptor encephalitis, Psychopharmacology, Paediatrics, Psychiatry

## Abstract

Autoimmune encephalitis (AE) can present like a psychiatric disorder. We aimed to illustrate the psychiatric manifestations, course and management of AE in a paediatric cohort. Neuropsychiatric symptoms, investigations and treatment were retrospectively retrieved in 16 patients (mean age 11.31, SD 2.98) with an AE diagnosis at the liaison psychiatry services in two UK tertiary paediatric centres. Psychiatric presentation was characterised by an acute polysymptomatic (predominantly agitation, anger outbursts/aggressiveness, hallucinations, and emotional lability) onset. Antipsychotics produced side effects and significant worsening of symptoms in four cases, and benzodiazepines were commonly used. This psychiatric phenotype should make clinicians suspect the diagnosis of AE and carefully consider use of treatments.

**Supplementary Information:**

The online version contains supplementary material available at 10.1007/s00406-021-01293-5.

## Background

Autoimmune encephalitis (AE) is a rare condition but increasingly recognised in children and adolescents [1]. Although considered to be underestimated, the incidence for antibody-mediated AE in paediatric patients is around 1.5 in a million [2] and it is a common cause of encephalopathy in this age population [3].

AE is a condition with high morbidity and recognised mortality [4]. The classic presentation of AE includes neurological, autonomic and psychiatric manifestations, such as confusion, agitation, psychotic features and mood fluctuations, and anti-NMDA receptor (NMDAR) encephalitis is the prototypical AE [5]. Diagnosis is often not considered early and the initial stages of AE can present as a psychiatric disorder, leading to longer duration of untreated illness and poorer outcomes [6]. Possible factors that might contribute to a delay in the diagnosis could stem from insufficient training about the range of presentations of autoimmune encephalitides amongst child psychiatrist, limited scope for investigation in mental health field and frame bias in clinical reasoning.

Therefore, prompt identification and initiation of immunotherapy are key clinical factors, and psychiatrists’ involvement within the multidisciplinary team is important to assist with diagnostic clarification and to offer their expertise in the use of psychotropic medications in this age group [7].

Consensus criteria for the diagnosis of anti-NMDAR encephalitis are widely used in adults [8] and they have a reasonably high sensitivity and specificity in children [9]. To detect as many cases as possible, the psychiatric presentation criterion includes broad symptoms with an acute onset.

In children that present with AE, the proposed consensus clinical criteria to suspect anti-NMDAR encephalitis was only 57% sensitive [10], and this observation has also recently been incorporated into the paediatric criteria [11]. As such many children with a polysymptomatic encephalopathy including psychiatric features are NMDAR antibody negative.

However, more detailed descriptions of psychiatric presentations in terms of phenomenology and course are needed to encourage psychiatrists to consider AE when formulating differential diagnoses. Recent studies have highlighted the complexity and heterogeneity of anti-NMDAR encephalitis psychopathology [12, 13]. Across all age groups (from 8 months to 84 years) severe agitation, psychotic features, speech abnormalities and catatonia were common reported psychiatric features in anti-NMDAR encephalitis and the association with neurological symptoms aided illness identification, but data analysis was not specific to paediatric population [13]. In paediatric populations, catatonia has been specifically analysed and the majority of patients manifested both hyperkinetic and hypokinetic features in the course of anti-NMDAR encephalitis [14]. Less is well known of the psychiatric symptoms of children presenting with AE, where no antibodies are identified [13, 15].

Here, we present a cohort of children with autoimmune encephalitis, reporting the psychiatric manifestations, course and management to inform on clinician´s knowledge in identifying potential AE diagnoses and increase competence in managing the psychiatric symptomatology.

## Method

Medical records of consecutive paediatric patients who were diagnosed with AE and referred to psychiatry liaison at two tertiary hospitals, between 2016 and 2019, were retrospectively reviewed. Patients were included based on the identification of neuronal surface associated with specific encephalitides in serum and/or cerebrospinal fluid (CSF) using immunofluorescence cell-based assays or immunoprecipitation assays in routine clinical use at the Oxford Autoimmune Neurology Diagnostic Laboratory [8]. Patients’ serum and/or CSF was investigated predominantly for the following antibodies: NMDAR, VGKC, LGI1, CASPR2, Glycine, GABAR, AMPAR. ANA, GAD and TPO antibodies were also tested.

Ab-negative AE patients were diagnosed based on established criteria [8] adapted to the paediatric population, since autoantibody profile seems to differ from adults [16], specifically, acute onset (less than 3 months) of encephalopathy or psychiatric symptoms, exclusion of an alternative cause, and at least two of the following inflammation indicators: characteristic neuroimaging changes, CSF pleocytosis, intrathecal oligoclonal band synthesis, characteristic electroencephalography (EEG) changes, definite response to immunotherapy.

Data extracted included demographics, medical and psychiatric past history, time of symptom onset, clinical presentation of AE with full psychiatric symptomatology and neurological manifestations, immunotherapy, psychiatric treatment and outcomes.

Psychiatric symptoms were grouped as per recognized symptom clusters: (1) behavioural changes (such as repetitive or stereotypical behaviours, agitation, aggressiveness, anger outbursts, personality changes, disinhibition, changes in eating and sleep patterns); (2) mood changes (emotional liability, anxiety, and irritability); (3) abnormal speech (echolalia, mutism); and (4) psychotic symptoms (delusion, hallucinations).

We used SPSS software to perform descriptive statistics. In addition, we conducted Chi-square test to investigate psychiatric symptoms differences (changes in behaviour, sleep disturbance, eating changes, mood symptoms, abnormal speech and psychotic symptom load) between patients with NMDAR antibody positive and NMDAR antibody negative AE.

## Results

### Basic demographic and past history

Thirteen females and three males with a mean age of 11.31 (median 11, range 4–15, SD 2.98) were referred.

Two patients (12.5%) had neurodevelopmental disorders (learning disabilities and autism) and none of them had previous psychiatric history. Two (12.5%) had a psychiatric family history and three (18.75%) had family history of autoimmune diseases (systemic lupus erythematosus, rheumatoid arthritis, and autoimmune thyroiditis).

Six (37.5%) presented with non-specific symptoms between 2 and 4 weeks before developing neuropsychiatric symptoms: four presented with non-specific viral symptoms, such as fever and sore throat, one presented with varicella, and another one suffered from a streptococcal throat infection. One case presented with Herpes simplex encephalitis 4 months before.

### Initial presentation

Seven patients (43.7%) initial presentation was mixed psychiatric and neurological symptoms, six (37.5%) first presented only with psychiatric symptoms and only three cases (18.8%) had a neurological onset. Initial psychiatric symptoms were abnormal behaviour (agitation/disinhibition, *n* = 11), emotional lability (*n* = 8), hallucinations (*n* = 8), speech changes (mutism/echolalia, *n* = 4), sleep problems (*n* = 3), anxiety (*n* = 2), and one had new onset of obsessive–compulsive symptoms.

### Psychiatric symptoms during the course of the illness

The three patients with only neurological symptoms presentation developed new psychiatric symptoms through the course of the illness. All patients had two or more psychiatric symptoms (for symptom details, see Tables 1 and 2). This was observed in both, NMDAR antibody positive and non-NMDAR antibody patients.

**Table 1:** Psychiatric features of paediatric AE

Symptom cluster	NMDAR + ve *N* = 7 (%)	NMDAR –ve *N* = 9 (%)	Total *N* = 16 (%)
Behavioural changes	7 (100)	8 (88.9)	15 (93.7)
Agitation	6 (85.7)	8(88.9)	14 (87.5)
Anger outbursts/aggressiveness	5 (71.4)	6 (66.7)	11(68.7)
Disinhibition	3 (42.8)	2 (22.2)	5 (31.2)
Personality changes/regression	4 (57.1)	3 (33.3)	7 (43.7)
Repetitive or stereotypical behaviours	3 (42.8)	1 (11.1)	4 (25)
Eating	2 (28.6)	4 (44.4)	6 (37.5)
Hyperphagia	1 (14.3)	0 (0)	1 (6.2)
Reduced appetite	1 (14.3)	4 (44.4)	5 (31.2)
Sleep patterns^a^	7 (100)	5 (55.5)	12 (75)
Insomnia	7 (100)	2 (22.2)	9 (56.2)
Hypersomnia	0 (0)	2 (22.2)	2 (12.5)
Sleep walking	1 (14.3)	1 (11.1)	2 (12.5)
Vivid dreams	0 (0)	2 (22.2)	2 (12.5)
Speech	5 (71.4)	4 (44.4)	9 (56.2)
Disorganized speech	2 (28.6)	1 (11.1)	3 (18.7)
Echolalia	2 (28.6)	0 (0)	2 (12.5)
Mutism	1 (14.3)	1 (11.1)	2 (12.5)
Pressure of speech	1 (14.3)	1 (11.1)	2 (12.5)
Mood symptoms	6 (85.7)	6 (66.7)	12 (75)
Irritability	1 (14.3)	2 (22.2)	3 (18.7)
Mood liability	4 (57.1)	4 (44.4)	8 (50)
Anxiety	1 (14.3)	2 (22.2)	3 (18.7)
Psychotic symptoms	5 (71.4)	8 (88.9)	13 (81.2)
Delusions	4 (57.1)	5 (55.5)	9 (56.2)
Paranoid ideas	3 (42.8)	4 (44.4)	7 (43.7)
Capgras	0 (0)	2 (22.2)	2 (12.5)
Grandiose ideas	1 (14.3)	0 (0)	1 (6.2)
Hallucinations	5 (71.4)	7 (77.8)	12 (75)
Visual hallucinations	4 (57.1)	6 (66.7)	10 (62.5)
Auditory hallucinations	4 (57.1)	3 (33.3)	7 (43.7)
Tactile hallucinations	0 (0)	1 (11.1)	1 (6.2)

^a^Sleep disturbance was statistically significant for NMDAR antibody positive AE

**Table 2:** Quantification of psychiatric clusters in AE

Number of symptom clusters	*N *total	*N* NMDAR +	*N* NMDAR –ve
Presence of the four main clusters (behavioural, speech, mood and psychotic features)	7/16	4/7	3/9
Presence of three main clusters	3/16	1/7	2/9
Presence of two main clusters	6/16	2/7	4/9
Presence of one main cluster	0/16	0/7	0/9

No significant differences in psychiatric symptoms between NMDAR antibody positive and NMDAR antibody negative AE

### Neurological symptoms during the course of the illness

Indistinctive of the initial presentation, all cases developed neurological symptoms as the illness evolved. All but four (75%) developed symptoms of delirium (disturbance of consciousness, reduced attention, disorientation). Notably some of these would constitute features of encephalopathy, more commonly adopted in neurology literature, but here we maintain the delirium construct of reporting symptoms. Neurological symptoms presented in Table 3.

**Table 3:** Neurological features of AE

Symptom cluster	*N*	% of total cases
Headache	2	12.5
Catatonia	4	25
Delirium	13	81.2
Choreoathetoid movements	1	6.2
Dystonia	3	18.7
Dyspraxia	2	12.5
Orofacial dyskinesia	1	6.2
Ocular myoclonus	1	6.2
Rigidity	4	25
Seizures	11	68.7
Short term memory impairment	10	62.5
Tremor	3	18.7
Weakness	1	6.2

### Investigations

Seven patients (44%) had CSF and serum NMDAR antibodies, with the other patients not having any neuronal surface antibodies identified. In this group three patient has evidence of high titres of anti-thyroid antibodies. In all cases, non-autoimmune disease mechanisms (infectious, metabolic, structural or inflammatory) were excluded.

Brain magnetic resonance imaging (MRI) showed hippocampal hyperintensity in three patients (18.7%) and the electroencephalography (EEG) demonstrated encephalopathy features in seven cases (43.7%). One patient (NMDAR + ve) was diagnosed with ovarian teratoma.

### Management

Five patients required admission to the Paediatric Intensive Care Unit (PICU) due to decreased level of consciousness. All the patients received immunotherapy. 14 were treated with corticosteroids, 13 with intravenous immunoglobulins (IVIG), and eight with plasma exchange (PLEX). Six patients required second-line therapies (rituximab).

13 also received psychotropic medications. The medications most used were benzodiazepines (lorazepam *n* = 10, and midazolam *n* = 2) and antipsychotics (risperidone *n* = 5, aripiprazole *n* = 4, haloperidol *n* = 4, olanzapine *n* = 2, quetiapine *n* = 1). Five patients were also on antihistamines (promethazine). Five cases were switched to another antipsychotic due to lack of improvement. Moreover, side effects requiring terminating medication were observed in four cases: dystonia with haloperidol (*n* = 2), worsening of neurological symptoms with risperidone (*n* = 1) and paradoxical reaction to lorazepam (*n* = 1).

The mean duration of hospitalization was 111.15 days (median 59, range 7, 457 days, SD 142.3), while the mean time until reincorporation to school was 134.14 days (median 65, range 37, 210, SD 58.42).

### Differences in psychiatric symptoms between anti-NMDAR encephalitis vs NMDAR antibody negative patients

The only difference between the psychiatric features between NMDAR positive and NMDAR negative group was the presence of sleep disturbances in patients with NMDAR antibody encephalitis [7/7 vs 5/9, chi square, *p* = 0.042]. There were no statistically significant differences in the rest of the psychiatric symptom load between NMDAR antibody positive and non-NMDAR patients (see Table 2).

## Discussion

The key observation in our study is that our paediatric cohort with AE consistently presented with a pattern of polysymptomatic psychiatric features. The most predominant were agitation (87%), hallucinations (75%), sleep pattern changes (75%) and mood changes (75%) which is consistent with previous studies in adults and children [17–21].

Recently, due to the high predominance of psychotic features in AE, an international consensus has suggested criteria to aid a rapid differential diagnosis to differentiate primary psychotic episodes from psychosis of autoimmune origin (autoimmune psychosis) characterised by isolated psychotic presentations with no, or minimal, neurological features [15]. Among our cohort with a psychiatric presentation, 13 developed abrupt psychotic symptoms and met all core proposed criteria to suspect an autoimmune aetiology (see Supplementary Material Table 1). This indicates that the criteria for adults may be applicable to paediatric population and, therefore, supporting the novel category of the so-called autoimmune psychosis.

Nevertheless, the critical question remains if early psychiatric symptoms sometimes occurring months before can be distinguishable from a primary psychiatric disorder. In this study, we found that children have numerous co-occurring psychiatric symptoms, all had two or more clusters of symptoms and 44% had the four clusters. This is uncommonly seen in children presenting with a primary psychiatric disorder [22]. Notably, only two children in our cohort have a neurodevelopmental co-morbidity commonly associated with childhood schizophrenia and psychosis and none had prior mental health history.

Patients in this cohort were managed to maximise environmental intervention, whilst minimising pharmacotherapy, particularly during the acute encephalopathy stage due to an increased intolerance to antipsychotic medication; specifically, the potential risk of worsening of AE symptoms and lower threshold for neuroleptic malignant syndrome (NMS) and other extrapyramidal symptoms. Therefore, it has been suggested that antipsychotic intolerance could be a warning sign to psychiatrists to suspect and investigate a diagnosis of anti-NMDAR encephalitis [23]. Moreover, lack of response of behavioural and psychiatric symptoms to psychotropic medication may mislead clinicians to continue increasing doses or switching antipsychotic perpetuating adverse risks and delaying appropriate treatment. In our sample, five cases were switched to another antipsychotic due to lack of improvement. Good psychiatric management can contribute to limiting extent of longer term damage [24]. We managed our patients, predominantly with benzodiazepines (lorazepam) and antihistamines (promethazine) with symptom response benefits without major side effects, as observed in another study of similar characteristics [25]. However, 69% of our sample was also treated with an antipsychotic.

This study has a number of limitations. First, the retrospective design with data collected from medical records being susceptible to varied observations, data available (including neurologic/psychiatric symptoms and detailed time of the specific features), synthesis of findings and records of medication administered and potential side effects. Second, systematic screening tools for psychiatric symptoms such as the present state examination were not used. Finally, included cases were obtained through referral to paediatric liaison psychiatry services which might also contribute to a high rate of unfavourable outcomes through a selection bias toward severe cases.

In summary, the presence of polysymptomatic psychiatric symptomatology, or in other words, overlapping symptom domains (particularly sleep disturbances), in a child presenting with an acute/sub-acute psychosis, in addition to the absence of other neurodevelopmental co-morbidities, poor cognitive function (e.g., deterioration in school performance), and poor or adverse reaction to anti-psychotics should alert the psychiatrist to an autoimmune aetiology, prior to onset of more recognisable neurological features. This is consistent with analyses of the adult literature.

This information may inform clinicians of the clinical features that should raise suspicion of AE, prompting further investigations and pragmatic treatment decisions to target the underlying cause of the psychiatric symptoms.

## Supplementary Information

Below is the link to the electronic supplementary material.

Supplementary file1 (DOCX 22 KB)
