# Supplementary material for: Characterizing the features and course of psychiatric symptoms in children and adolescents with autoimmune encephalitis
Source: Eur Arch Psychiatry Clin Neurosci. 2021 Jul 17;272(3):477–82. doi: 10.1007/s00406-021-01293-5 (PMC8938365; doi:10.1007/s00406-021-01293-5)
Supplement: Supplementary file 1 — Supplementary file1 (DOCX 22 KB) [file 406_2021_1293_MOESM1_ESM.docx]

**Supplementary Material Table 1:** Clinical features of paediatric patients for the diagnosis of possible autoimmune psychosis based on consensus diagnostic criteria by Pollak et at., 2019.

| **Proposed diagnostic criteria for autoimmune psychosis** | **1** | **2** | **3** | **4** | **5** | **6** | **7** | **8** | **9** | **10** | **11** | **12** | **13** |
| --- | --- | --- | --- | --- | --- | --- | --- | --- | --- | --- | --- | --- | --- |
| **Meet criteria for a diagnosis of probable Autoimmune psychosis** | **Y** | **Y** | **Y** | **Y** | **Y** | **Y** | **Y** | **Y** | **Y** | **Y** | **Y** | **Y** | **Y** |
| Abrupt onset (rapid progression of psychotic symptoms <3 months) | **+** | **+** | **+** | **+** | **+** | **+** | **+** | **+** | **+** | **+** | **+** | **+** | **+** |
| Currently or recently diagnosed with a tumour | **-** | **-** | **-** | **-** | **-** | **-** | **-** | **-** | **-** | **+** | **-** | **-** | **-** |
| Movement disorder (catatonia or dyskinesia) | **-** | **-** | **+** | **+** | **-** | **-** | **-** | **+** | **-** | **-** | **+** | **-** | **-** |
| Adverse response to antipsychotics, raising suspicion of NMS (rigidity, hyperthermia or raised creatine kinase) * | **NR** | **NR** | **NR** | **NR** | **NT** | **+** | **NT** | **NR** | **NT** | **NR** | **+** | **NT** | **NR** |
| Severe or disproportionate cognitive dysfunction | **+** | **-** | **+** | **+** | **+** | **+** | **-** | **+** | **-** | **+** | **+** | **+** | **-** |
| A decreased level of consciousness | **+** | **+** | **+** | **+** | **-** | **+** | **+** | **+** | **+** | **+** | **+** | **+** | **+** |
| The occurrence of seizures that are not explained by a previously known seizure disorder | **+** | **+** | **+** | **+** | **-** | **-** | **+** | **-** | **+** | **+** | **+** | **-** | **+** |
| A clinically significant autonomic dysfunction (abnormal or unexpectedly fluctuant blood pressure, temperature, or heart rate) ** | **NR** | **NR** | **NR** | **NR** | **NR** | **NR** | **NR** | **NR** | **NR** | **+** | **NR** | **NR** | **NR** |

Three patients are not included in the table were as they did not present with psychotic symptoms

Abbreviations: NMS: Neuroleptic Malignant Syndrome, NT: No treatment; NR: Not reported

* adverse response likely not to be recorded if negative, therefore NR could be negative

** autonomic dysfunction often poorly recorded
